# Relapsed/refractory multiple myeloma: standard of care management of patients in the Gulf region

**DOI:** 10.46989/001c.137860

**Published:** 2025-05-08

**Authors:** Ahmad Alhuraiji, Khalil Al Farsi, Kayane Mheidly, Hesham Elsabah, Honar Cherif, Anas Hamad, Mahmoud Marashi, Hussni Al Hateeti, Hani Osman, Mohamad Mohty

**Affiliations:** 1 Department of Hematology, Kuwait Cancer Control Center, Kuwait; 2 Department of Hematology, Sultan Qaboos University Hospital, Muscat, Oman; 3 Department of Medicine, Division of Hematology, Sheikh Shakhbout Medical City, Abu Dhabi, UAE; 4 Department of Hematology and Bone Marrow Transplantation, National Centre for Cancer Care and Research (NCCCR), Hamad Medical Corporation, Doha, Qatar; 5 Pharmacy Department, National Center for Cancer Care and Research (NCCCR), Hamad Medical Corporation, Doha, Qatar; 6 Mediclinic City Hospital and Dubai Hospital, Dubai, UAE; 7 Department of Hematology, Tawam Hospital, Al Ain, Abu Dhabi, United Arab Emirates; 8 Sorbonne University, Department of Clinical Hematology and Cellular Therapy, Saint-Antoine Hospital, AP-HP, INSERM UMRs 938, Paris, France

**Keywords:** Multiple myeloma, daratumumab, relapsed/refractory, Gulf, high risk

## Abstract

Clinical management of patients with relapsed/refractory multiple myeloma (RRMM) can be challenging, whereby each relapse is associated with progressively poorer outcomes. In addition, changes in disease biology and patient characteristics hamper treatment strategies in this setting, as do toxicities accumulated across previous lines of therapy. The availability of several new treatment classes has brought about improvements in outcomes, but with median survival in the RRMM setting at only ~32 months, a review of current standard of care treatments and considerations for optimizing care in this setting is warranted. Here, we discuss our preferred approach to treating patients with RRMM, based on our collective experience across the Gulf region. We present position statements for the treatment of lenalidomide-sensitive and -refractory patients, as well as for those patients experiencing late relapse. We discuss the major impact that anti-CD38 agents daratumumab and isatuximab have had on the management of RRMM, which is reflected in our preferred use of daratumumab-based regimens across the lenalidomide-sensitive and -refractory settings. For late-relapse settings, we discuss how bispecific antibodies and chimeric antigen receptor [CAR]-T cells are among the biggest breakthroughs in recent years, achieving excellent responses in triple-class exposed patients. While the use of these agents is not yet widespread in the Gulf region, we advocate their use where available and discuss strategies to manage and minimize common toxicities and adverse events associated with their use.

## 1. Introduction

Multiple Myeloma (MM) is a B-cell malignancy defined by plasma cell infiltration of the bone marrow and end-organ damage, attributable to the classic features of hypercalcemia, renal insufficiency, anemia, and bone lesions (CRAB criteria).^1^ Despite tremendous advances in the clinical management of MM, it remains incurable and is characterized by a relapsing disease course and progressive genetic alterations of tumor cell subpopulations within an immunosuppressive bone marrow environment.^1,2^ Relapse and disease progression often occur even when patients have initially experienced complete remission, and can be identified by the presence of specific biochemical and clinical changes in relation to the initial treatment.^3^ Patients are defined as having relapsed/refractory MM (RRMM) when they have achieved at least a minimal response to frontline treatment, but subsequently become non-responsive or progress within 60 days of the last therapy.^3–5^

Management of RRMM can be challenging,^6^ and outcomes in the RRMM setting become progressively worse with each relapse, in keeping with the most effective treatments being used in the earliest treatment lines.^7^ Treating RRMM is further complicated by changes in both the biological heterogeneity of the disease (e.g., aggressiveness of relapse) and in patient characteristics (e.g., age/frailty, new comorbidities over time).^3,6,8^ Clonal evolution and cytogenetic abnormalities can also have a major impact on disease at relapse, with the proportions of patients with high-risk markers including del17p, t(4;14), and 1q21+ tending to increase, potentially complicating disease management and leading to resistance to specific treatment classes.^6,9–13^ A further consideration is the accumulation of treatment-related toxicities (e.g., peripheral neuropathy, cardiotoxicity), which can become a limiting factor on the choice of future therapies.^14^

The advent of several new classes of drugs has helped to increase median survival rates in MM to around 8 years, particularly in younger fit patients^15,16^; however, in the RRMM setting, median survival is only ~32 months,^17^ and is often substantially lower especially in patients who are triple class exposed (TCE).^18,19^ Current frontline SOC includes continuous maintenance with lenalidomide,^20,21^ meaning the vast majority of patients who relapse are lenalidomide-refractory, a group with poorer outcomes than lenalidomide-sensitive patients.^22^ As such, it is important to optimize treatments for RRMM, and distinguishing treatment strategy on the basis of sensitivity to lenalidomide is an important factor.^23^

Here, we present position statements for treatment of RRMM in the Gulf region, based on our own clinical experience in this setting. We present positions for lenalidomide-sensitive, lenalidomide-refractory, and late-relapse patients, and discuss the use of traditional and newer agents in these settings, including proteasome inhibitors, anti-CD38 monoclonal antibodies, and immunotherapy (bispecific antibodies [BsAbs] and chimeric antigen receptor [CAR]-T cells) for late-relapse disease. Finally, we highlight some of the common toxicities observed with immunotherapy and discuss strategies to minimize and manage BsAb-associated adverse events (AEs).

## 2. Materials and Methods

A group of seven experts from across the Gulf region of the Middle East convened twice in June 2024. Those attendees who were able to, joined the meeting physically in Doha, Qatar, and others joined virtually. The discussion was moderated by one international independent expert from France (Dr M. Mohty). The expert group comprised seven hematologists and one pharmacist who were selected due to their recognized seniority and expertise in the management of MM. During these meetings the expert group collectively discussed and agreed upon position statements for treatment of RRMM.

## 3. Lenalidomide-sensitive RR patients

### 3.1. Review of key evidence

Lenalidomide has been a cornerstone of MM treatment over the last decade^24^ and is used as part of most frontline triplet or quadruplet regimens as well as in continuous maintenance regimens.^20,21^ It is therefore very uncommon to treat lenalidomide-sensitive RRMM patients in the current era. However, there are a minority of patients who are lenalidomide-sensitive, including those who have been treated historically with autologous stem cell transplant (ASCT) and lenalidomide-free regimens, who are only now experiencing their first relapse. In these lenalidomide-naïve patients, the best option is to use lenalidomide-based regimens for relapse.

There are several options in the RRMM setting that utilize a lenalidomide-dexamethasone (Rd) backbone, and typically exclude bortezomib which is also commonly used in frontline treatment. These Rd-based regimens include the proteasome inhibitors carfilzomib (KRd) and ixazomib (Ixa-Rd), the immunostimulatory monoclonal antibody elotuzumab (Elo-Rd), as well as more recently the anti-CD38 daratumumab (Dara-Rd), all of which are recommended in lenalidomide-sensitive RRMM in international guidelines.^20,21^

The ASPIRE phase 3 study investigated KRd vs. Rd in patients with RRMM who had received 1–3 prior treatments (<20% lenalidomide), and reported a median progression-free survival (PFS) of 26.3 vs. 17.6 months (hazard ratio [HR]: 0.69; p=0.0001), with overall survival (OS) rates at 24 months of 73.3% and 65%, respectively.^25^ Response rates were also improved with KRd, with 87.1% patients achieving a partial response or greater compared with 66.7% in the Rd arm; nearly a third (31.8%) of patients receiving KRd achieved a complete response (CR) or better compared with only 9.3% receiving Rd. After a long-term median follow-up of 67.1 months, a longer median OS of ~8 months was reported with KRd (48.3 vs. 40.4 months with Rd), but this benefit was not observed in those with high-risk cytogenetic abnormalities.^26^ The results from ASPIRE support the use of triplet therapy in lenalidomide-sensitive RRMM, but the rate of serious AEs was higher in the KRd compared with the Rd group (65.3% vs. 56.8%) as were certain grade ≥3 AEs including cardiac failure (4.3% vs. 2.1%), ischemic heart disease (3.8% vs. 2.3%) and hypertension (6.4% vs. 2.3%),^25,26^ that would warrant caution of its use among patients at risk of adverse cardiac outcomes.

The all-oral Ixa-Rd regimen was investigated in the phase 3 TOURMALINE-MM1 trial, in RRMM patients having received 1–3 prior treatments who were not refractory to lenalidomide.^27^ After a median 14.7-month follow-up, median PFS was significantly longer in patients receiving Ixa-Rd than Rd alone (20.6 vs. 14.7 months; HR 0.74, p=0.01), and this benefit was observed in all pre-specified subgroups including elderly patients and those with high-risk cytogenetic abnormalities.^27^ Response rates were also improved with triplet therapy, with 48% of patients in the Ixa-Rd group achieving CR or very good partial response (VGPR), compared with 39% in the Rd group. However, final analysis at the long-term follow-up reported that the key secondary endpoint of increased OS with Ixa-Rd vs. Rd was not met (53.6 vs. 51.6 months after a median follow-up of ~7 years).^28^

Elo-Rd was compared with Rd in the phase 3 ELOQUENT-2 trial in patients with RRMM who had received 1–3 prior therapies (5–6% previous lenalidomide).^29^ Median PFS in the Elo-Rd arm was 19.4 months vs. 14.9 months with Rd (HR 0.70; p<0.0001), and the overall response rate (ORR) was 79% vs. 66%, respectively.^29^ The benefits of adding elotuzumab to Rd were maintained across most prespecified subgroups, including elderly patients (≥65 years) and those with high-risk cytogenetic abnormalities (particularly del17p). Final OS analysis of the ELOQUENT-2 trial reported an 8.7 month increase in median OS with Elo-Rd vs. Rd after a minimum 70.6 months follow-up, confirming the triplet as a treatment option in the RRMM setting.^30^

Dara-Rd was compared with Rd in the phase 3 POLLUX trial in lenalidomide-sensitive patients with RRMM who had received ≥1 previous line of therapy.^31^ At primary analysis, the 12-month PFS rate was 83.2% with Dara-Rd vs. 60.1% with Rd (HR 0.37; p<0.001). The ORR was also significantly higher with Dara-Rd (92.9% vs. 76.4%), with the clinical benefits observed across all subgroups.^31^ The extended follow-up of POLLUX (median 44.3 months) demonstrated a median PFS in the Dara-Rd group of 44.5 months vs. 17.5 months with Rd, and minimal residual disease negativity was 30.4% vs. 5.3%, respectively.^32^ While cross-trial comparisons must be undertaken with caution, the median PFS achieved with Dara-Rd was unprecedented,^32^ reflecting the major impact of daratumumab in the management of MM.^33^ In agreement, final OS results from the POLLUX trial showed a significant improvement in median OS of 15.8 months longer with Dara-Rd vs. Rd alone.^34^

The key trials in lenalidomide-sensitive RRMM patients and their primary outcomes are summarized in **Table 1**.

**Table 1.** Key trials in lenalidomide-sensitive RRMM patients

Trial phase/name (Registration number)	Comparison / trial design	Patient N	Primary endpoint results	Primary reference
Phase 3 ASPIRE (NCT01080391)	KRd vs. Rd (<20% prior R)	792	PFS: median (KRd vs. Rd) 26.3 months, vs. 17.6 months; HR 0.69, p=0.0001	*Stewart et al., 2015^25^*
Phase 3 TOURMALINE-MM (NCT01564537)	Ixa-Rd vs. Rd (non–R-refractory)	722	PFS: median (Ixa-Rd vs. Rd) 20.6 months vs. 14.7 months; HR 0.74, p=0.01	*Moreau et al., 2016^27^*
Phase 3 ELOQUENT-2 (NCT01239797)	Elo-Rd vs. Rd (5–6% prior R)	646	PFS: median (Elo-Rd vs. Rd) 19.4 months vs. 14.9 months; HR 0.70, p<0.0001	*Lonial et al., 2015^29^*
Phase 3 POLLUX (NCT02076009)	Dara-Rd vs. Rd R-sensitive	569	PFS: median (Dara-Rd vs. Rd) NE vs. 18.4 months; HR 0.37, p<0.001	*Dimopoulos et al., 2016^31^*

d, dexamethasone; Dara, daratumumab; Elo, elotuzumab; HR, hazard ratio; Ixa, ixazomib; K, carfilzomib; NE, not estimable; PFS, progression-free survival; R, lenalidomide; RRMM, relapsed/refractory multiple myeloma

### 3.2. Position statement: Lenalidomide-sensitive RRMM patients

The following is our recommended position statement for SOC treatment in this setting:

**Dara-Rd** would be the treatment of choice, particularly for elderly patients.

This is a rare patient group and lenalidomide could be used in these patients.

### 3.3. Expert clinical opinion

**Figure 1.**
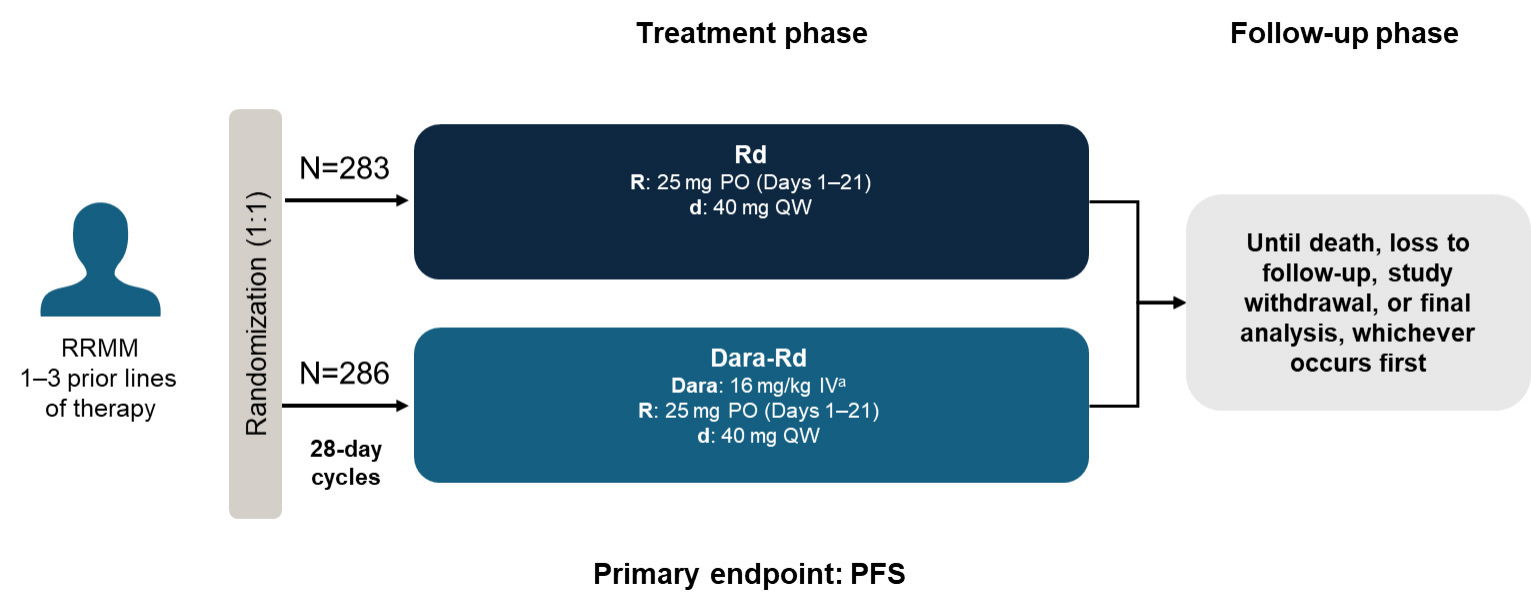
Study design of the phase 3 POLLUX trial in lenalidomide-sensitive RRMM patients ^a^Days 1, 8, 15 and 22 (first 2 cycles only) then every 2 weeks (cycles 3–6) and every 4-weeks (from cycle 7) thereafter. d, dexamethasone; Dara, daratumumab; IV, intravenous; PFS, progression-free survival; PO, orally; QW, once weekly; R, lenalidomide; RRMM, relapsed or refractory multiple myeloma.

Developing the optimal treatment strategy after relapse is more challenging than in frontline MM, and often upon reassessment, patients may have developed new comorbidities, frailty, and additional cytogenetic abnormalities. After reviewing frontline medication, it is increasingly rare to find a patient who is lenalidomide-sensitive but in such cases, using an Rd backbone is the best option. Our first choice would be Dara-Rd, particularly for patients who are elderly or frail but with good renal function. This is based on the available evidence, mainly the results from the POLLUX trial (study design in **Figure 1**),^31^ but is further broadly supported by a network meta-analysis of 22 clinical trials in RRMM.^35^ Although not restricted to lenalidomide-sensitive patients, anti-CD38-based regimens (i.e., daratumumab- and isatuximab-based) were identified in the meta-analysis to be the most effective agents in RRMM by ORR.^35^ However, isatuximab alongside Rd has not been examined in RRMM outside of a phase 1b study,^36^ and as such there is limited evidence for its use in this setting.

In some patients, KRd may be a good second option, although its use has to be implemented with caution due to the increased risk of cardiovascular events with carfilzomib, particularly given the high rates of metabolic syndrome among patients in the Gulf region.^37,38^ We are aware of some local protocols where carfilzomib-based regimens are used in patients who are considered high risk at relapse, although there are no randomized clinical trials supporting its use in this setting. In our opinion, Dara-KR would be a reasonable option in this case, as would Dara-KRd. Ixazomib is not extensively used in the Gulf region for RRMM, and despite the generally accepted benefits of having an all-oral regimen, we have experienced some negative effects associated with this. Such effects would have been avoided with a controlled dose in the clinic setting, and points to caution in patients with potential age-related cognitive impairment.

Another consideration in lenalidomide-sensitive RRMM patients would be a second ASCT at time of relapse. This may be appropriate if the patient is young and fit with an existing source of stem cells and may be warranted given the patient would not have been receiving maintenance therapy. Historically, this was considered an option if the patient had been in remission for over two years, but we suggest that a four-year remission cutoff may be more appropriate. A two-year relapse would now be considered an early relapse, and likely place the patient into the high-risk category, for whom a different treatment strategy would be warranted.

## 4. Lenalidomide-refractory RRMM patients

### 4.1. Review of key evidence

The majority of RRMM patients are considered lenalidomide-refractory, but this is a broad group encompassing many different potential scenarios, each requiring a different approach. For example, transplant-eligible patients may have received ASCT then progressed on lenalidomide maintenance, or transplant-ineligible patients may have initially received bortezomib plus Rd (VRd) then progressed while receiving lenalidomide maintenance.

If patients are only lenalidomide-refractory, then based on the available phase 3 evidence, it is generally standard practice to switch to an anti-CD38, plus either a proteasome inhibitor or pomalidomide, with dexamethasone.^20,21^ Most approved regimens commonly used today in this setting are triplet-based, with anti-CD38 agents added to doublets (e.g., bortezomib/carfilzomib-dexamethasone or pomalidomide-dexamethasone). The use of doublet-based regimens is mainly restricted to very old and/or frail patients.^20,21^

In the phase 3 CANDOR trial, daratumumab combined with Kd (Dara-Kd) was compared to Kd only in RRMM (patients receiving 1–3 prior lines of therapy).^39^ Median PFS in the overall patient population was 28.6 months with Dara-Kd vs. 15.2 months with Kd only (HR 0.59).40 Importantly, this PFS benefit was maintained in the lenalidomide-refractory (28.1 vs. 11.1 months; HR 0.46) as well as the lenalidomide-sensitive (28.6 vs. 19.9 months; HR 0.63) population.^40^ Furthermore, there was consistent benefit in median PFS with Dara-Kd compared with Kd alone in the lenalidomide-refractory population, regardless of the number of prior lines of therapy (1 prior line, 25.0 vs. 9.3 months; ≥2 prior lines, 28.1 vs. 12.0 months). In addition, the Dara-Kd regimen had clinical benefit in the proteasome inhibitor-refractory population, likely reflecting the high use of bortezomib relative to carfilzomib in upfront treatments.^40^ It is important to note that in the CANDOR trial, carfilzomib was given twice weekly, but in practice it is common to give once weekly, as it has been shown that this is equally as safe and effective as twice weekly dosing across different settings.^41–43^

Daratumumab was also examined alongside bortezomib-dexamethasone (Dara-Vd) in the phase 3 CASTOR trial, which compared Dara-Vd to Vd alone in RRMM patients with ≥1 previous line of therapy.^44^ After 3 years, Dara-Vd demonstrated a significantly better PFS than Vd alone (median PFS 16.7 vs. 7.1 months; HR 0.31) in the overall population.^45^ This PFS benefit was maintained in patients who were refractory to lenalidomide (median PFS 7.8 vs. 4.9 months; HR 0.22) and in those receiving previous bortezomib treatment (median PFS 12.1 vs. 6.7 months; HR 0.35), confirming its potential utility in the RRMM setting.

The IKEMA trial investigated isatuximab alongside Kd (Isa-Kd) vs. Kd alone and had a very similar trial design to CANDOR.^46^ In the overall population, after a median follow-up of 44 months, median PFS was 41.7 months in the Isa-Kd arm and 20.8 months in the Kd arm (HR 0.59).^47^ This benefit was observed in lenalidomide-refractory (HR 0.59) and -sensitive (HR 0.56) patients, but not in patients with prior proteasome inhibitor treatment (HR 0.82).^47^

Isatuximab was combined with a pomalidomide-dexamethasone backbone (Isa-Pd) in the ICARIA-MM phase 3 trial in patients with RRMM who received ≥2 prior lines of therapy.^48^ After a median 11.6 months follow-up, patients treated with Isa-Pd had a median PFS of 11.5 months vs. 6.5 months in patients treated with Pd alone (HR 0.596).^48^ These findings were maintained in subgroup analyses of patients who were refractory to lenalidomide (HR 0.59) proteasome inhibitor (HR 0.58), or both (HR 0.58), confirming its utility across the range of RRMM settings. However, the median PFS in this trial was notably shorter than in trials using the Kd backbone, and it could be speculated that this is due to a potential class-resistance to immunomodulatory agents occurring in some, but not all, lenalidomide-refractory patients. In keeping with this, a shorter median PFS was also observed in the phase 3 APOLLO trial in RRMM where daratumumab was combined with the Pd backbone (Dara-Pd) to give a median PFS of 12.4 months compared with 6.9 months in the Pd only group (HR 0.63), with similar results observed in lenalidomide-refractory patients (9.9 vs. 6.5 months).^49^

Pomalidomide has also been evaluated alongside bortezomib-dexamethasone (PVd) in the phase 3 OPTIMISMM trial, where RRMM patients had a median PFS of 11.2 months compared with 7.1 months in patients treated with Vd alone (HR 0.61).^50^ In this trial, improvements in PFS were observed in both lenalidomide-refractory (17.8 vs. 9.5 months) and lenalidomide-sensitive (22.0 vs. 12.0 months) patients, as well as in those who had received prior bortezomib (17.8 vs. 12.0 months), meaning PVd is another potential option in this setting. Additional options in the RRMM lenalidomide-refractory setting include elotuzumab alongside a Pd backbone (Elo-Pd), which showed benefit over Pd alone in the phase 2 ELOQUENT-3 trial, including in patients refractory to both lenalidomide and proteasome inhibitors.^51^

In summary, there are a range of treatments in the lenalidomide-refractory MM setting that are recommended in international guidelines,^20,21^ typically including daratumumab- (e.g., Dara-Pd, Dara-Vd, Dara-Kd) and isatuximab-based regimens (e.g., Isa-Pd, Isa-Kd), but also extending to other regimens that are non-anti-CD38-based, including PVd and Elo-Pd.

The key trials in lenalidomide-refractory RRMM patients and their primary outcomes are summarized in **Table 2**.

**Table 2.** Key trials in lenalidomide-refractory RRMM patients

Trial phase/name (Registration number)	Comparison / trial design	Patient N	Primary endpoint results	Primary reference
Phase 3 CANDOR (NCT03158688)	Dara-Kd vs. Kd (1–3 prior lines of therapy)	466	PFS: median (Dara-Kd vs. Kd) NR vs. 15.8 months; HR 0.63, p=0.0027	*Dimopoulos et al., 2020^39^*
Phase 3 CASTOR (NCT02136134)	Dara-Vd vs. Vd (≥1 previous line of therapy)	498	PFS: median (Dara-Vd vs. Vd) NR vs. 7.2 months; HR 0.39, p<0.001)	*Palumbo et al., 2016^44^*
Phase 3 IKEMA (NCT03275285)	Isa-Kd vs. Kd (1–3 prior lines of therapy)	302	PFS: median (Isa-Kd vs. Kd) NR vs. 19.2 months; HR 0.53, p=0.0007	*Moreau et al., 2021^46^*
Phase 3 ICARIA-MM (NCT02990338)	Isa-Pd vs. Pd (≥2 prior lines of therapy)	307	PFS: median (Isa-Pd vs. Pd) 11.5 months vs. 6.5 months; HR 0.596, p=0.001	*Attal et al., 2019^48^*
Phase 3 APOLLO (NCT03180736)	Dara-Pd vs. Pd (≥1 previous line of therapy)	304	PFS: median (Dara-Pd vs. Pd) 12.4 months vs 6.9 months; HR 0.63, 2-sided p=0.0018	*Dimopoulos et al., 2021^49^*
Phase 3 OPTIMISMM (NCT01734928)	PVd vs. Vd (1–3 prior lines of therapy)	281	PFS: median (PVd vs. Vd) 11.2 months vs. 7.1 months; HR 0.61, p<0.0001	*Richardson et al., 2019^50^*
Phase 2 ELOQUENT-3 (NCT02654132)	Elo-PD vs. Pd (≥1 previous line of therapy)	117	PFS: median (Elo-PD vs. Pd) 10.3 months vs. 4.7 months; HR 0.54, p=0.008	*Dimopoulos et al., 2018^51^*

d, dexamethasone; Dara, daratumumab; Elo, elotuzumab; HR, hazard ratio; Isa, isatuximab; K, carfilzomib; NR, not reached; P, pomalidomide; PFS, progression-free survival; R, lenalidomide; RRMM, relapsed/refractory multiple myeloma; V, bortezomib

### 4.2. Position statement: Lenalidomide-refractory RRMM patients

The following is our recommended position statement for SOC treatment in this setting:

***Dara-Kd*** (KdD) – first choice.

If an anti-CD38 regimen is not available/cannot be used, a carfilzomib approach would be used: **KCd** (where C is cyclophosphamide), or **KPd** (where P is pomalidomide) would be regimens of choice

### 4.3. Expert clinical opinion

**Figure 2.**
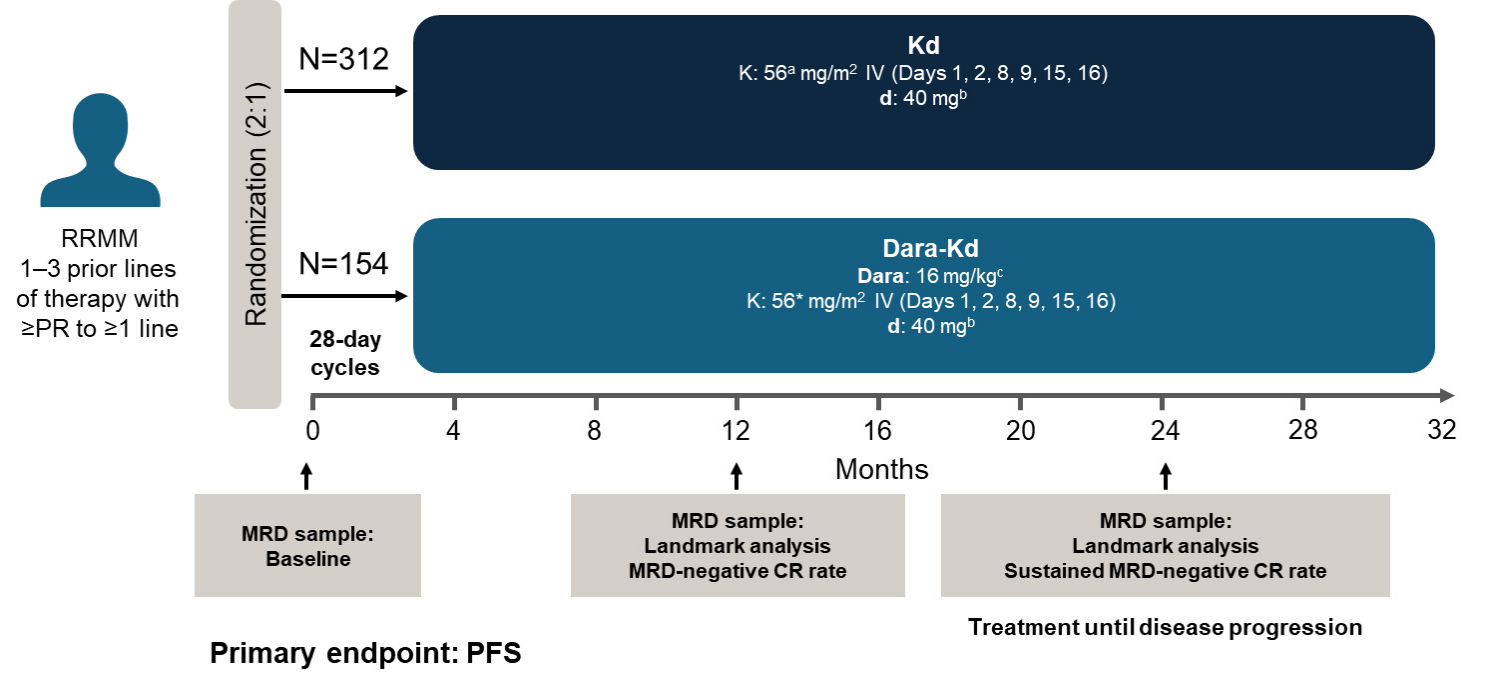
Study design of the phase 3 CANDOR trial in lenalidomide-refractory RRMM patients ^a^Carflizomib 20 mg/m2 administered on days 1 and 2 of cycle 1; ^b^Every week starting from week 2; ^c^First dose is 8 mg/kg split over two days followed by 16 mg/kg weekly (first 2 cycles only) then every 2 weeks (cycles 3–6) and every 4-weeks (from cycle 7) thereafter. CR, complete response; d, dexamethasone; Dara, daratumumab; IV, intravenous; K, carfilzomib; MRD, minimal residual disease; PFS, progression-free survival; RRMM, relapsed or refractory multiple myeloma.

In our experience, using an anti-CD38–based regimen in the lenalidomide-refractory setting is appropriate and effective. Dara-Kd is our recommended first choice, based primarily on the results of the phase 3 CANDOR trial (study design in **Figure 2**) in the lenalidomide-refractory population, regardless of the number of prior lines of therapy.^40^ There is vast experience in using subcutaneous daratumumab in the Gulf region, while isatuximab not as widely available and its use mainly restricted to specific cases of interest. Other daratumumab-based regimens that could be used are Dara-Pd and Dara-Vd, but they are less preferable than Dara-Kd, due to potential treatment resistance (e.g., class resistance encompassing pomalidomide, and bortezomib-resistance due to its widespread use in frontline regimens).

Our second choice (in the case of poor access to anti-CD38 agents) would be to use another carfilzomib-based regimen, such as KCd, which can be a good option when the patient has limited insurance coverage, due to cyclophosphamide being relatively inexpensive. Alternatively, KPd or PVd (if the patient is not bortezomib-refractory) are additional second options. However, we would tend to avoid pomalidomide if possible due to potential class resistance within the lenalidomide-refractory population, and the fact that patients tend to have better outcomes upon class switching.^52^ When using pomalidomide-based regimens, we have often found that reducing the dose from 4 mg to 2 or 3 mg daily is necessary to limit neutropenia, which is common among Arab populations.^53^

We highlight that of these second options, PVd is the only regimen that is phase 3 evidence-based, and while we are aware that KCd and KPd are commonly used in the lenalidomide-refractory setting, the evidence base supporting their use is more limited.^54–57^ Managing potential carfilzomib-based cardiotoxicity is a consideration due to its effects on the myocardium and cardiac vasculature.^58^ Monitoring for hypertension, low-density lipoprotein, and troponin or B-type natriuretic peptide levels are all important, as is collaboration with a cardio-oncologist to perform regular echocardiograms during treatment. Cardiotoxicities with carfilzomib are likely more severe than with bortezomib due to the irreversible nature of proteasome inhibition with carfilzomib,^58^ and reducing carfilzomib from twice weekly to once weekly dosing can help reduce cardiotoxicities.^42,43^

## 5. Position statement: Late-relapse RRMM patients

### 5.1. Review of key evidence

SOC in the frontline setting is currently triplet therapy with singlet or doublet maintenance, thus it is becoming increasingly common at first or second relapse for patients to be TCE or refractory to immunomodulatory agents, proteasome inhibitors, and anti-CD38 agents. TCE patients who are penta-refractory have very poor outcomes, with median OS as low as 6 months,^18^ and until recently were only considered for palliative care. However, in the last few years, several new classes of treatment have become available, including belantamab mafodotin (anti-drug conjugate), and selinexor (XPO1 inhibitor), as well as immunotherapy (CAR T-cells and BsAbs), which are among the biggest breakthroughs in RRMM treatment to date.^59,60^

BsAbs link the malignant plasma cell to the T-cell through the CD3 complex, leading to destruction of the plasma cell through effector cell activation.^60^ There are several B-cell antigens, with B-cell maturation antigen (BCMA) among the most common due to its high specificity and high expression on malignant plasma cells. Several agents are approved and recommended for use in later-line RRMM, including teclistamab and elranatamab (both targeting BCMA), and talquetamab (targeting GPRC5D).^20^

**Figure 3.**
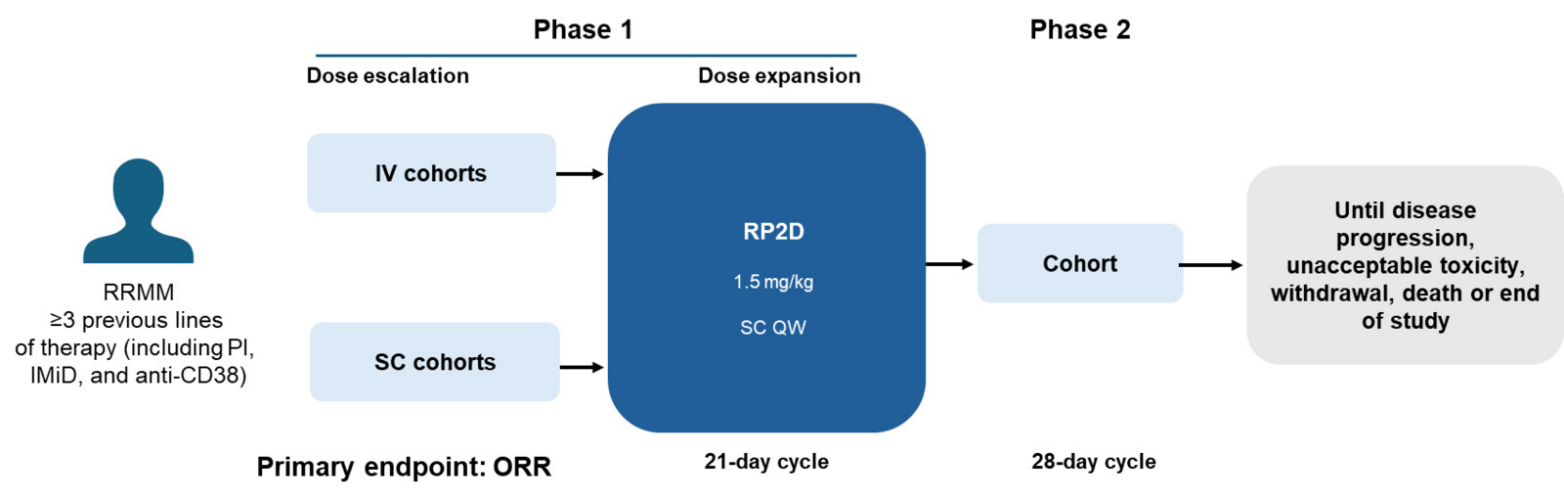
Study design of the phase 1/2 MajesTEC-1 trial in late-relapse RRMM patients IMiD, immunomodulatory drug; IV, intravenous; ORR, overall response rate; PI, proteasome inhibitor; QW, weekly; RP2D, recommended phase II dose; RRMM, relapsed/refractory multiple myeloma; SC subcutaneous.

Teclistamab was approved by the US FDA in 2022,^61^ based on the result of the phase 1/2, non-randomized MajesTEC-1 trial in 165 heavily pre-treated patients with RRMM who were TCE (78% triple class refractory; Figure 3).^62^ At the primary analysis, the ORR was 63.0% with the majority of responders achieving a VGPR or greater (59.4%), and 45.5% patients achieving a CR or greater.^62^ Although there are caveats in making cross-trial comparisons, it is of note that the ORR reported in MajesTEC-1 was almost double the ORR achieved by belantamab mafodotin in the DREAMM-2 phase 2 trial in triple class refractory patients.^63^ At longer follow-up (median 2 years), median PFS in MejesTEC-1 was 12.5 months, and OS was 21.9 months.^64^ While the ORR was substantially lower in patients with extramedullary disease, stage 3 disease, and those with high tumor burden, it was higher in patients who had received ≤3 previous lines of treatment.^62^ Among non-hematologic AEs, the safety profile included a high incidence (72%) of cytokine release syndrome (CRS), although cases were generally mild, and five cases (3%) of immune effector cell-associated neurotoxicity syndrome (ICANS), all of which were grade 1/2. Arguably most important was the likelihood of increased infections, which were not only present at a high incidence (78%), but many (52%) were grade 3 or 4 in severity.^63^ The increased incidence of infections is likely due to a combination of factors, including patients being immunosuppressed due to dexamethasone treatment, the effects of BCMA skewing the immune system towards a single antigen, and the destruction of the few remaining healthy BCMA-expressing plasma cells. Thus, the drastic reduction in immunoglobulin production alongside potential T-cell exhaustion after weekly teclistamab injections likely creates conditions for opportunistic infections.

Elranatamab is another BCMA-targeted BsAb that has been approved^65^ in TCE patients based on the results of the open-label, non-randomized MagnetisMM-3 phase 2 trial.^66^ In the cohort of 123 patients with no prior BCMA-directed treatment, the median previous line of therapies received was five, with an upper age range of 89 years (median 68 years), reflecting the suitability of older patients for BsAb therapy.^66^ The ORR was 61% with 35% of patients achieving a CR or higher^66^; similar to teclistamab in the MajesTEC-1 trial,^62^ the ORR was substantially lower in MagnetisMM-3 patients with extramedullary disease, worse disease stage, and penta-refractory disease. After a median follow-up of 14.7 months, median PFS was 17.2 months and median OS 21.9 months,^66^ similar to those observed in MajesTEC-1.^64^ The safety profile of elranatamab was similar to that of teclistamab, with a high incidence of CRS and infections.^66^

Talquetamab is a third BsAb option in late-relapse RRMM patients,^67^ and was investigated in the phase 1/2 MonumenTAL-1 trial.^68^ Patients had a median six lines of prior therapy (including previous BsAbs), which included 79% patients with triple-class refractory disease.^68^ The ORR was 74% and 73% in the talquetamab 0.4 mg once weekly and 0.8 mg every two weeks treatment groups, respectively. In patients with prior T-cell redirection therapy, the ORR dropped to 44.4% in patients who had received prior BsAbs.^69^ The safety profile included a high incidence (>70%) of CRS, with infections slightly lower than those seen with teclistamab and elranatamab, albeit frequent enough to require careful management. Additional AEs that were more frequent with talquetamab include dysgeusia and skin/nail lesions, both of which can negatively impact patient quality of life and occurred in over half of patients in the MonumenTAL-1 trial.^68,69^

The key trials in late-relapse RRMM patients and their primary outcomes are summarized in **Table 3**.

**Table 3.** Key trials in late-relapse RRMM patients

Trial phase/name (Registration number)	Comparison / trial design	Patient N	Primary endpoint results	Primary reference
Phase 1/2 MajesTEC-1 (NCT03145181& NCT04557098)	Teclistamab (≥3 previous lines of therapy)	165	ORR: 63.0% (65 patients [39.4%] had ≥CR)	*Moreau et al., 2022^62^*
Phase 2 MagnetisMM-3 (NCT04649359)	Elranatamab (Refractory to ≥1 IMiD, ≥1 PI and ≥1 anti-CD38)	123	ORR: 61.0% (75 patients [35.0%] had ≥CR)	*Lesokhin et al., 2023^66^*
Phase 1/2 MonumenTAL-1 (NCT03399799)	Talquetamab (0.4/0.8 mg) (Heavily pre-treated including ≥1 IMiD and ≥1 PI)	232	ORR^a^: 0.4 mg dose, 70.0% (≥CR: 23%); 0.8 mg dose, 64% (≥CR: 23%)	*Chari et al., 2022^68^*

^a^Primary endpoint was safety, ORR shown for consistency with other trials in table

CR, complete response; IMiD, immunomodulatory drug; ORR, overall response rate; PI, proteasome inhibitor; RRMM, relapsed/refractory multiple myeloma

### 5.2. Position statement: Late-relapse RRMM patients

The following is our recommended position statement for SOC treatment in this setting:

**Bispecific antibody or CAR T-cell therapy** is the first choice for all eligible TCE patients. Selinexor should only be considered when there is no access to BsAbs/CAR T.

Of note, to date there is very little experience with BsAbs in the region, and CAR T is prohibitively expensive and not available in all countries in the region.

### 5.3. Expert opinion

Despite limited experience with BsAbs or CAR T for RRMM among physicians in the Gulf region, we are of the consensus that patients with late-relapse RRMM who are TCE should be considered for BsAbs or CAR T if available. Our current opinion is based primarily on the published evidence base, with only very few patients having been treated with BsAbs in our clinics to date. Generally, we would give an intravenous infusion of immunoglobulin before initiating treatment, as well as antibiotic, anti-fungal and anti-viral medications to combat potential infections. Of note, a meta-analysis of 36 studies including 1560 patients, reported that the rate of CRS is higher with CAR T-cell therapy than with BsAbs (88% vs. 59%),^70^ with more events of grade ≥3 (7% vs. 2%), which should be taken into consideration when weighing up the benefit–risk of each type of immunotherapy.

A potential consideration when treating with BsAbs is that BCMA is overexpressed in MM and can be shed from the cell surface into circulation, where it may potentially act as a decoy for teclistamab. Reduction of the tumor load, through pretreatment with selinexor, for example, before treating with BsAbs may be a consideration. Another method to potentially improve outcomes with BsAbs is to use conventional chemotherapy which not only helps shrink highly proliferating tumors, but also creates tumor lysis that releases immune-reactive components to help activate the BsAb immune synapse.^71,72^

In late-relapse cases where there is no access to BsAbs, or indeed when a patient has relapsed after BsAbs, then selinexor-based regimens may be considered. However, we would normally use selinexor at a lower dose than the 80 mg twice weekly used in the phase 3 STORM trial,^73^ for example 40 or 60 mg twice weekly, or even 60 mg once weekly to allow for gradual dose increases to ensure adequate tolerability. In our experience, strong supportive care is needed with selinexor including prophylactic anti-emetics to counteract gastrointestinal toxicity, as well as thrombopoietin receptor agonists and granulocyte-colony stimulating factor to manage cytopenia. Alternative options to selinexor include belantamab mafodotin-based^74,75^ and venetoclax-based regimens. Although venetoclax was associated with increased mortality due to infections in RRMM patients in the phase 3 BELLINI trial,^76^ recent results suggest it could play a role in the treatment of RRMM patients with t(11:14), which can indicate an overexpression of BCL-2 protein.^77^ In general, we would not recommend allotransplant for late-relapse, due to burden of hospitalization and risk of infection, followed by progression usually within a few months.

#### 5.3.1. Managing toxicities with immunotherapy in RRMM

For teclistamab, elranatamab, and talquetamab, the prescribing information includes details of pre-medication (including paracetamol, acetaminophen, anti-histamines, and dexamethasone) and step-up dosing protocols to help minimize toxicities.^61,65,67^ Education of nurses on potential toxicities of these newer agents is also important. The International Myeloma Working Group (IMWG) has published specific guidance on how to manage CRS associated with BsAbs, including recommendations for grade 3/4 events to be managed with tocilizumab and high-dose steroids in the intensive care unit.^78^ IMWG guidelines also specify guidelines for ICANS management, mainly centered on the use of dexamethasone with addition of methylprednisone for more severe cases.^78^ Key to any BsAb-based treatment regimen is a strategy for managing potential infections. Firstly, the majority of RRMM patients have hypogammaglobulinemia,^79^ hence every patient should be supplemented with intravenous immunoglobulin, although the amount and frequency administered will vary by case. This approach is supported by a post-hoc analysis of the MagnetisMM-3 trial, where the rates of all infections (bacterial, fungal, viral, and unspecified) were lower in those with vs. without immunoglobulin replacement therapy, including those infections that were grade ≥3.^80^ Secondly, a combination of prophylactic anti-infective agents is recommended, and there are published consensus recommendations from global experts for the monitoring, prophylaxis, and treatment of infections in patients receiving BsAbs,^81^ as well as specific recommendations from the IMWG.^78^ Should a patient develop a fever, we recommend being extremely fastidious in identifying the responsible microorganism prior to treatment. We recommend the same level of vigilance as would be given for an allotransplant such that everyone is on high alert for serious infections. Finally, to minimize risk of infection, if a patient is in CR or VGPR, then we would follow label recommendations to switch to fewer monthly administrations, which would also minimize T-cell exhaustion, kill less healthy plasma cells, and reduce treatment costs. One clear exception to this would be patients with a high tumor burden, where excess plasma BCMA can potentially reduce the activity of BsAbs.

## 6. Discussion and conclusion

While the clinical management of RRMM remains challenging, additions to the treatment armamentarium have brought about welcome improvements in this setting. Anti-CD38 agents have had a major impact across lenalidomide-sensitive and -refractory patients, and physicians across the Gulf region have excellent experience in their use, particularly with daratumumab. Immunotherapy has shown tremendous promise in late-relapse, triple-class exposed patients, and while its use is still limited in the Gulf region, its increased adoption will undoubtedly further improve outcomes in RRMM.

### Authors’ Contribution

All authors made a significant contribution to the work reported, whether that was in the conception, acquisition of data, analysis and interpretation, or took part in drafting, revising, critically reviewing the article, and gave final approval of the version to be published, have agreed on the journal to which the article has been submitted, and agree to be accountable for all aspects of the work.

### Competing of Interest – COPE

Ahmad Alhuraiji has nothing to declare

Khalil Al Farsi has nothing to declare

Hussni Al Hateeti has nothing to declare

Hesham Elsabah has nothing to declare

Honar Cherif has nothing to declare

Anas Hamad has nothing to declare

Mahmoud Marashi has nothing to declare

Kayane Mheidly has nothing to declare

Hani Osman has nothing to declare

Mohamad Mohty declares honoraria from Adaptive Biotechnologies, Amgen, Astellas Pharma, Bristol-Myers Squibb, GlaxoSmithKline, Janssen Cilag EMEA, Jazz Pharmaceuticals, Medac Pharma Inc., Novartis, OncoPep, Inc., Pfizer, Sanofi, Takeda Oncology, and Therakos.

### Ethical Conduct Approval – Helsinki – IACUC

Not applicable.

### Informed Consent Statement

Not applicable.

### Data Availability Statement

Not applicable.
